# Association of obesogenic environment and hedonic appetite with chronic fatigue in Turkish adults

**DOI:** 10.3389/fnut.2025.1660721

**Published:** 2025-10-13

**Authors:** Gizem Helvacı, Fatma Tayhan

**Affiliations:** ^1^Faculty of Health Sciences, Department of Nutrition and Dietetics, Mehmet Akif Ersoy University, Burdur, Türkiye; ^2^Faculty of Health Sciences, Department of Nutrition and Dietetics, Çankırı Karatekin University, Çankırı, Türkiye

**Keywords:** chronic fatigue, exposome, hedonic appetite, obesogenic environment, Turkish adults

## Abstract

**Background:**

Chronic fatigue is a persistent state of physical, emotional, and cognitive exhaustion that does not resolve with rest. Behavioral and environmental factors may contribute to the onset and course of chronic fatigue. In this context, we aimed to examine the predictive roles of obesogenic environment and hedonic appetite in chronic fatigue.

**Method:**

We conducted the study on 505 Turkish adults aged 18–65. Participants completed a questionnaire form containing questions about demographic characteristics, dietary habits, the Chalder Fatigue Scale (CFS), the Power of Food Scale (PFS), and the Assessment of Obesogenic Environment Assessment Scale (AOES).

**Results:**

The proportion of women is higher in the third tertile (highest fatigue) than in the first tertile (*p* = 0.014). The proportion of those who exercise regularly in the third tertile is lower compared to the first and second tertiles (*p* = 0.001). The percentage of participants who are sedentary for more than 4 h per day is lower in the first tertile than in the second and third tertiles (*p* = 0.007). The proportion of individuals who often eat in front of a screen is higher in the third tertile (41.2%) than in the first tertile (27.2%; *p* = 0.016). The proportion of individuals in the first tertile of fatigue level who prefer fast food when eating out is 44.5%, which is lower than that of the second and third tertiles (*p* < 0.05). As hedonic appetite (β = 0.370, *p* < 0.001) and exposure to obesogenic environment (β = 0.131, *p* = 0.002) levels increase, fatigue symptoms also increase. It was found that individuals with high levels of chronic fatigue had a more obesogenic environment in terms of physical, social, and economic aspects; however, they were environmentally advantaged in terms of cultural factors and access to experts (*p* < 0.05). Participants who self-report having a regular sleep schedule exhibit significantly lower levels of fatigue (β = −0.146, *p* < 0.001).

**Conclusion:**

Our findings show that obesogenic environments, irregular sleep schedules, and hedonic appetite are associated with fatigue perception and may serve as its predictors. Strategies to improve physical, social, and economic aspects of obesogenic environments may help prevent chronic fatigue, while addressing hedonic appetite through psychotherapy could also be beneficial. Effective prevention and management of chronic fatigue can strengthen long-term societal resilience and support overall wellbeing.

## Introduction

Obesogenic environments refer to aspects of the social environment that contribute to the prevalence of obesity by influencing dietary habits and physical activity levels. Obesogenic factors include the density of fast-food restaurants, the availability of processed food products, food advertising, and social norms (1). Additionally, endocrine-disrupting chemicals are among the factors that contribute to an obesogenic environment. Among these chemicals known as obesogens are phthalates and bisphenol A, which are found in food packaging and plastic containers, as well as pesticides used in agriculture. Obesogens can alter insulin sensitivity and disrupt metabolism and endocrine regulation, resulting in an increase in both the number and size of adipocytes (2). Environmental aspects have strong effects on health and disease development over time, as they contribute to a dietary intake high in energy, fat, and sugar and to metabolic processes that increase body adiposity. Furthermore, the obesogenic environment may exacerbate chronic inflammation, which is important in the pathophysiology of obesity-related diseases (3).

The concept of the exposome has emerged in order to holistically understand the environmental factors that contribute to obesity and other health problems (4). This concept encompasses both external and internal factors, including climate change, air pollution, the urban environment, green spaces, the home environment, lifestyle, pesticides, medications, access to food, economic status, the microbiome, and inflammation (5). The evaluation of the exposome, which refers to the lifelong effects of various risk factors on human health, is very challenging and is therefore generally investigated at specific time points. Achieving a complete assessment of the exposome is currently unattainable, yet even partial evaluations still yield valuable information (6). In this study, the evaluation of particular obesogenic environmental factors among community-dwelling adults is expected to provide meaningful contributions to the field of exposome research.

Environmental conditions can significantly direct individuals' eating behaviors and lead to hedonic nutrition. Hedonic eating refers to the increase in the urge to eat and the loss of control over eating due to the sensory properties of food and social experiences, despite the absence of physical hunger (7). It stimulates the brain's reward system, leading to the frequent and excessive consumption of tasty foods (8). The obesogenic environment can promote hedonic consumption because individuals have easier access to unhealthy food options (9, 10). This non-homeostatic eating pattern leads to an increase in body weight and changes in the neural systems that control behavior. Over time, one becomes accustomed to energy-dense foods, and the cycle that leads to the development of obesity continues (11).

The effects of factors such as the obesogenic environment and hedonic appetite are not limited to metabolic outcomes but can also shape an individual's overall perception of fatigue. Fatigue is defined as a decrease in performance as a result of physical, emotional, and mental exertion. It manifests after periods of activity with physiological changes and improves with rest. However, sometimes, independent of effort, a lack of motivation, a decrease in physical and cognitive capacity can be observed, and quality of life is negatively affected (12). This type of fatigue is chronic. Fatigue symptoms that do not improve with rest and develop without a clear cause, accompanied by memory and concentration deficiencies, are referred to as “chronic fatigue” (13). It has been reported that individuals with chronic fatigue adopt a healthy lifestyle less frequently (14). Unhealthy lifestyle and obesity have also been associated with fatigue and reduced functionality (15). Evidence shows that the interaction between lifestyle and fatigue is bidirectional. The etiology of fatigue perception that does not improve with rest is complex. It can be caused by the interaction of multiple factors, including genetics, the immune system, psychological conditions, adrenal systems, sleep, and nutrition (16). Eating a healthy diet, having a healthy environment, and regulating environmental factors are possible. The aim of this study is to examine the association of modifiable risk factors, particularly the obesogenic environment and hedonic food intake, with symptoms of chronic fatigue in community-dwelling adults.

*Research questions*

1. Do adults' fatigue levels differ significantly according to their demographic characteristics and lifestyle habits?
2. Do adults' fatigue levels differ significantly according to their dietary habits?
3. Is there a significant relationship between adults' exposure to obesogenic factors, hedonic appetite, and fatigue levels?
4. What are the variables that predict adults' fatigue levels?

## Method

The study is cross-sectional and was conducted with 505 adults living in Turkey between January and May 2025. According to a sensitivity analysis conducted using G^*^Power software (*N* = 505, α = 0.05, 1-β = 0.80, *m* = 7), the minimum detectable effect size in the multiple linear regression model was found to be *f*^2^ ≈ 0.029 (*R*^2^ ≈ 0.029). This result indicates that the sample size is sufficient to detect small but meaningful effects.

Those who volunteered to participate, had internet access, were literate enough to understand the survey, and were between the ages of 18 and 65 were included. Those who are pregnant, breastfeeding, have metabolic disorders, or have been diagnosed with other medical conditions that could confound the results were not included in the study. The online survey prepared in Turkish was distributed through social media platforms using the snowball sampling method. In the first section of the questionnaire, information about the study was provided, and individuals who checked the consent box voluntarily participated in the study. The survey includes sections on participants' demographic characteristics and dietary habits, as well as the Chalder Fatigue Scale, the Nutritional Power Scale, and the Obesogenic Environment Assessment Scale for Adults.

### Chalder Fatigue Scale (CFS)

Initially developed as a 14-item scale to assess the severity of fatigue, the scale was later revised to an 11-item version (17, 18). The Turkish validity and reliability study was conducted by Adin et al. in 2022 (19). The scale includes two sub-dimensions: physical fatigue (CFS-PF) and mental fatigue (CFS-MF). The physical fatigue subdimension assesses physical symptoms such as lack of energy, muscle weakness, malaise, fatigue, somnolence, and difficulty initiating daily activities (items 1–7). In contrast, the mental fatigue subdimension evaluates cognitive symptoms, including difficulties with attention and concentration, slips of the tongue during speech, trouble finding the appropriate word, and memory problems (items 8–11). The total score ranges from 0 to 33. Low scores reflect a lower level of fatigue. Cronbach's α values were reported as 0.862 for CFS-PF, 0.704 for CFS-MF, and 0.863 for the total CFS score, respectively (19).

### The Power of Food Scale (PFS)

The scale was developed by Lowe et al. as a 15-item measure to assess hedonic hunger (20). The Turkish validity and reliability study was conducted by Ulker et al. in 2021 (21). The Turkish version of the scale consists of 13 items on a 5-point Likert type and is divided into three sub-dimensions (food available, food present, and food taste). Individuals' enjoyment of eating, their tendency to seek food at unexpected times, and the intensity of their thoughts about food are measured through the food available subdimension. The food present subdimension encompasses the desire to eat triggered by seeing or smelling food, difficulty in refraining from consuming favorite high-calorie foods, constantly contemplating delicious foods, and the inclination to consume certain foods even if they are harmful. The food taste subdimension includes the excitement and anticipation experienced before tasting a preferred food, a strong focus on flavor while eating, interest in foods recommended or described by others, and the desire for foods to be maximally tasty and enjoyable. Items are rated from 1 to 5 (strongly disagree: 1, disagree: 2, neutral: 3, agree: 4, strongly agree: 5). Three sub-dimensions and the total scale score are calculated by summing the item scores and dividing by the number of items. Both subscale scores and the total scale score range from 1 to 5. An increase in the score indicates a higher tendency toward hedonic hunger. The Cronbach's alpha for the PFS scale was calculated as 0.922; the corresponding values for the “food available,” “food present,” and “food taste” subscales were reported as 0.849, 0.797, and 0.82 (21).

### The Assessment of Obesogenic Environment Scale (AOES)

The scale aims to evaluate the environment that leads to the development of obesity in individuals. Developed by Kesik and Saglam (22), a validity and reliability study was conducted in Turkish. It is a 7-point Likert type and contains 32 items. It consists of four sub-dimensions: factors and opportunities related to the physical environment, cultural determinants and access to experts, social determinants and their effects, and economic determinants and their effects. The sub-dimension regarding factors and opportunities related to the physical environment consists of 10 items, with the 7th and 8th items being reverse scored. They examine access to healthy foods, meal services, gyms, safe walking areas, and the influence of long work or study hours. The sub-dimension of cultural determinants and access to experts evaluates the social, cultural, and economic factors that influence individuals' healthy eating and physical activity behaviors. This sub-dimension consists of a total of 10 items, with only the 7th item being scored directly, while all other items are reverse scored. It covers the presence of active individuals in one's social circle, cultural attitudes, media and popular figures influence, access to professionals, affordability and availability of healthy foods and equipment, and exposure to unhealthy food advertisements. The sub-dimension of social determinants and their effects evaluates individuals' healthy eating and physical activity behaviors in the context of their social environment, family, peer groups, societal perceptions, and media influence. This sub-dimension consists of a total of eight items, with only the 6th item being reverse scored. The items cover factors such as body image perceptions shaped by social media, the view that healthy eating is boring, attitudes of family and friends toward physical activity, the influence of societal discourse, eating habits at home, the effect of internet use on physical activity, and the food options provided in educational or work environments. The economic determinants and effects sub-dimension consists of four items, and none of the items are reverse-scored. This sub-dimension covers factors such as the affordability of healthy foods and sports equipment, access to paid physical activity opportunities, and the impact of financial constraints on healthy lifestyle choices. It is scored between 32 and 224. Low scores indicate healthy factors, while high scores indicate obesogenic factors. In the validation and reliability study of the scale, the test-retest Cronbach's alpha value was reported as 0.815, and the intraclass correlation coefficient was 0.687. The Cronbach's alpha for internal consistency was 0.761. These results demonstrate that the scale has good reliability, moderate test-retest consistency, and very good internal consistency (22).

### Statistical analysis

The IBM SPSS (Statistical Package for the Social Sciences) 25.0 package program was used to evaluate the data. The normality of the data distribution was assessed by examining skewness and kurtosis coefficients, as well as by performing the Shapiro–Wilk test. Parametric tests were used in the analyses because the variables met the normality assumption. Mean, standard deviation, number, and percentage values were used for descriptive statistics. Because the Chalder Fatigue Scale lacks a cutoff point for the total score, participants were divided into tertiles based on their scores. This method, commonly used in scales without a standard cutoff point, was chosen to compare participants with low, moderate, and high fatigue levels. General characteristics, scale scores, and dietary habits were analyzed according to these tertiles using the chi-square test and one-way ANOVA. Different superscript letters indicate statistically significant differences between tertiles based on the Bonferroni *post-hoc* test (*p* < 0.05). Tertiles sharing at least one common letter do not differ significantly from each other. The associations between scale scores, age, and body mass index were assessed using the Pearson correlation analysis. Multiple linear regression analysis was used to explore the predictors of fatigue scores. Significance was evaluated at the *p* < 0.05 level.

## Results

Table 1 presents the distribution of scale scores and general characteristics of the participants according to tertiles of fatigue level. As fatigue tertiles increased, the total PFS score and its sub-dimensions—food present and food taste—showed a statistically significant progressive increase, with each tertile differing significantly from the others (*p* < 0.001 for all, Bonferroni *post-hoc*). For food availability, *post-hoc* comparisons indicated that tertile 1 scored significantly lower than both tertile 2 and tertile 3, while no significant difference was observed between Tertiles 2 and 3. The total AOES score in the third tertile according to fatigue level is higher than in the first and second tertiles (*p* < 0.001). *Post-hoc* comparisons indicated that, for AOES-P and AOES-E, tertile 1 scored significantly lower than both tertile 2 and tertile 3, while no significant difference was observed between tertiles 2 and 3. For AOES-S, tertile 3 had significantly higher scores than tertile 1 and 2. Participants in the first tertile of the fatigue scale had significantly higher AOES-C scores than those in the second and third tertiles (*p* < 0.001). Regarding age, *post-hoc* analysis revealed that participants in Tertile 1 were significantly older than those in tertile 3, while tertile 2 did not differ significantly from either group (*p* = 0.035). When examining the sex distribution according to the level of fatigue, the proportion of women in the third tertile is higher than in the first tertile (*p* = 0.014). The proportion of those who exercise regularly in the third tertile is lower compared to the first and second tertiles (*p* = 0.001). In the first tertile, the proportion of people who are sedentary for more than 4 h per day is lower than in the second and third tertiles (*p* = 0.007). In the third tertile, the percentage of people who self-reported having a regular sleep schedule is lower than in the first and second tertiles (*p* < 0.001). In other variables (smoking, alcohol consumption, age, BMI), no statistically significant difference was found according to the level of fatigue (*p* > 0.05).

**Table 1:** Distribution of scale scores and general characteristics according to individuals' fatigue level tertiles.

**Variables**	**Chalder fatigue scale**			***p*** **-value** ^a^
	**Tertile 1 (0–10) (*****n*** = **173)**	**Tertile 2 (11–16) (*****n*** = **162)**	**Tertile 3 (17–33) (*****n*** = **170)**	
	***X*** ±**SD**	***X*** ±**SD**	***X*** ±**SD**	
**PFS total score**	2.48 ± 1.01^a^	2.94 ± 0.86^b^	3.34 ± 0.93^c^	< 0.001^**^
PFS-Food available	2.31 ± 1.03^a^	2.80 ± 0.98^b, c^	3.05 ± 1.09^c^	< 0.001^**^
PFS-Food present	2.52 ± 1.08^a^	2.94 ± 0.92^b^	3.39 ± 0.99^c^	< 0.001^**^
PFS-Food taste	2.60 ± 1.15^a^	3.09 ± 0.95^b^	3.56 ± 1.04^c^	< 0.001^**^
**AOES total score**	119.18 ± 21.66^a, b^	123.77 ± 17.47^b^	129.06 ± 19.64^c^	< 0.001^**^
AOES-P	37.06 ± 12.14^a^	40.79 ± 10.02^b, c^	42.81 ± 10.97^c^	< 0.001^**^
AOES-C	43.09 ± 14.84^a^	38.39 ± 13.89^b, c^	37.68 ± 13.44^c^	< 0.001^**^
AOES-S	24.92 ± 10.42^a, b^	27.45 ± 9.06^b^	30.28 ± 10.08^c^	< 0.001^**^
AOES-E	14.09 ± 7.68^a^	17.13 ± 7.13^b, c^	18.28 ± 7.69^c^	< 0.001^**^
Age	28.03 ± 11.6^a^	26.04 ± 8.60^a, c^	25.41 ± 8.63^c^	0.035^*^
BMI (kg/m^2^)	23.83 ± 4.65	23.15 ± 4.09	23.20 ± 4.20	0.272
	***n*** **(%)**	***n*** **(%)**	***n*** **(%)**	***p*** **-value** ^b^
**Sex**				
Male	60 (34.7)^a^	47 (29.0)^a, b^	35 (20.6)^b^	0.014^*^
Female	113 (65.3)^a^	115 (71.0)^a, b^	135 (79.4)^b^	
**Smoking habit**				
Yes	42 (24.3)	40 (24.7)	42 (24.7)	0.995
No	131 (75.7)	122 (75.3)	128 (75.3)	
**Alcohol use**				
Yes	29 (16.8)	35 (21.6)	36 (21.2)	0.463
No	144 (83.2)	127 (78.4)	134 (78.8)	
**Exercise regularly**				
Yes	60 (34.7)^a^	48 (29.6)^a^	30 (17.6)^b^	0.001^*^
No	113 (65.3)^a^	114 (70.4)^a^	140 (82.4)^b^	
**Daily sedentary time**				
< 1 h	10 (5.8)^a^	6 (3.7)^a^	4 (2.4)^a^	0.007^*^
1–2 h	50 (28.9)^a^	24 (14.8)^b^	28 (16.5)^b^	
3–4 h	55 (31.8)^a^	56 (34.6)^a^	57 (33.5)^a^	
>4 h	58 (33.5)^a^	76 (46.9)^b^	81 (47.6)^b^	
**Self-reported regular sleep schedule**				
Regular	88 (50.9)^a^	68 (42.0)^a^	52 (30.6)^b^	< 0.001^**^
Irregular	85 (49.1)^a^	94 (58.0)^a^	118 (69.4)^b^	

^*^p < 0.05, ^**^p < 0.001.

^a^One-way ANOVA.

^b^Pearson's chi-squared.

The presence of different superscript letters (^a, b, c^) indicates significant differences among groups.

BMI, body mass index; PFS, power food scale; AOES, The Assessment of Obesogenic Environment Scale; AEOS-P, Factors regarding physical environment and opportunities; AOES-C, Cultural determinants and access to experts; AOES-S, Social determinants and their effects; AOES-E, Economic determinants and their effects; Tertile 1, lowest fatigue; Tertile 3, highest fatigue.

Table 2 shows the distribution of participants' eating habits according to their levels of fatigue. The proportion of individuals who often eat in front of a screen is higher in the third tertile (41.2%) than in the first tertile (27.2%; *p* = 0.016). The proportion of individuals in the first tertile of fatigue level who prefer fast food when eating out is 44.5%, which is lower than that of the second and third tertiles (*p* < 0.05). When eating out, the proportion of those who prefer home-cooked style meals is higher in the first tertile compared to the third tertile (*p* < 0.05). The proportion of individuals who consider themselves to have a healthy diet is higher in the first tertile (23.7%) than in the third tertile (12.9%; *p* = 0.005). Skipping main meals, purchasing food based on advertisements, and the frequency of craving sweets did not show a statistically significant difference according to the level of fatigue (*p* > 0.05).

**Table 2:** Distribution of individuals' eating habits according to their levels of fatigue.

**Variables**	**Chalder fatigue scale**						***p-*value**
	**Tertile 1 (0–10) (*****n*** = **173)**		**Tertile 2 (11–16) (*****n*** = **162)**		**Tertile 3 (17–33) (*****n*** = **170)**		
	***n***	**%**	***n***	**%**	***n***	**%**	
**Skipping main meals**							
Yes	99	57.2	103	63.6	116	68.2	0.106
No	74	42.8	59	36.4	54	31.8	
**Eating in front of screen**							
Never	14^a^	8.1	3^b^	1.9	7^a, b^	4.1	0.016^*^
Rarely	32^a^	18.5	23^a^	14.2	29^a^	17.1	
Sometimes	80^a, b^	46.2	79^b^	48.8	64^a^	37.6	
Often	47^a^	27.2	57^a, b^	35.2	70^b^	41.2	
**Food purchase due to ads**							
Never	69	39.9	44	27.2	50	29.4	0.126
Rarely	53	30.6	58	35.8	61	35.9	
Sometimes	42	24.3	54	33.3	47	27.6	
Often	9	5.2	6	3.7	12	7.1	
**Food choice when eating out**							
Fast food	77^a^	44.5	96^b^	59.3	107^b^	62.9	0.005^*^
Home-cooked	25^a^	14.5	14^a, b^	8.6	7^b^	4.1	
Salads	6^a^	3.5	4^a^	2.5	4^a^	2.4	
Grilled/meat dishes	65^a^	37.6	48^a^	29.6	52^a^	30.6	
**Craving for sweets**							
Rarely	69	39.9	51	31.5	60	35.3	0.093
Sometimes	79	45.7	87	53.7	72	42.4	
Almost daily	25	14.5	24	14.8	38	22.4	
**Perceived healthy eating**							
No	49^a^	28.3	42^a^	25.9	72^b^	42.4	0.005^*^
Partially	83^a^	48.0	86^a^	53.1	76^a^	44.7	
Yes	41^a^	23.7	34^a, b^	21.0	22^b^	12.9	

^*^p < 0.05. Chi-square test used for categorical comparisons across fatigue tertiles. The presence of different superscript letters (^a, b, c^) indicates significant differences among groups.

Tertile 1: lowest fatigue, Tertile 3: highest fatigue.

**Figure 1:**
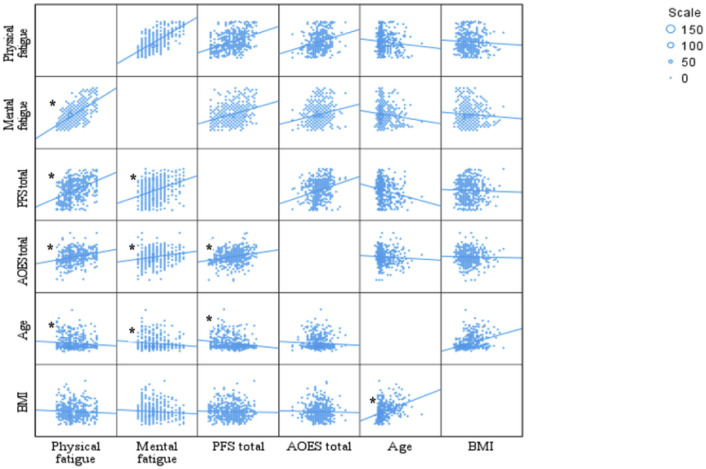
Correlations between scales, age, and BMI. *p < 0.05. Pearson Correlation test. PFS, power food scale; AOES, The Assessment of Obesogenic Environment Scale; BMI, body mass index.

Figure 1 summarizes the relationships among individuals' scale scores, age, and BMI. Accordingly, the food power scale is positively correlated with mental fatigue (*r* = 0.333, *p* < 0.001) and physical fatigue (*r* = 0.401, *p* < 0.001) scores. Additionally, the food power scale scores increase as AOES scores increase (*r* = 0.291, *p* < 0.001). AOES scores are significantly positively correlated with both physical fatigue scores (*r* = 0.282) and mental fatigue scores (*r* = 0.219; *p* < 0.001). A significant negative correlation was found between age and the total PFS score (*r* = −0.175, *p* < 0.001). In addition, a negative correlation was observed between age and mental fatigue score (*r* = −0.124, *p* = 0.005), as well as between age and physical fatigue score (*r* = −0.096, *p* = 0.032). A positive correlation was found between age and BMI (*r* = 0.346, *p* < 0.001).

In Table 3, the results of the multiple linear regression analysis conducted with the fatigue level (CFS total score) as the dependent variable are presented. The model is statistically significant (Adj. *R*^2^ = 0.242, *p* < 0.001) and explains 24.2% of the total variance. The total PFS score and the total AOES score are significantly and positively correlated with the CFS score. In other words, as the level of hedonic food craving (β = 0.370, *p* < 0.001) and exposure to an obesogenic environment (β = 0.131, *p* = 0.002) increase, the symptoms of fatigue also increase. In contrast, self-reporting having a regular sleep schedule significantly reduces the CFS score (β = −0.146, *p* < 0.001). Variables such as age, BMI, sex, and regular exercise were not found to be significant predictors of fatigue levels (*p* > 0.05).

**Table 3:** Multiple linear regression, with the CFS score as a dependent variable.

**Independent variable**	***B***	**SE**	**β**	***t***	***p-*value**	**%95 CI**
PFS total score	2.714	0.309	0.370	8.774	< 0.001^**^	2.106/3.321
AOES total score	0.048	0.015	0.131	3.167	0.002^*^	0.018/0.078
BMI (kg/m^2^)	−0.060	0.074	−0.035	−0.809	0.419	−0.205/0.085
Age (year)	0.000	0.033	0.001	0.015	0.988	−0.064/0.065
Sex	−0.329	0.699	−0.020	−0.471	0.638	−1.702/1.044
Self-reported regular sleep schedule	−2.190	0.606	−0.146	−3.614	< 0.001^**^	−3.380/−0.999
Exercise regularly	−1.332	0.687	−0.080	−1.939	0.053	−2.681/0.017
	Dependent variable: CFS, Adj. *R*^2^: 0.242 *n*: 505 *p*: < 0.001^**^					

^*^p < 0.05, ^**^p < 0.001.

BMI, body mass index; PFS, power food scale; AOES, The Assessment of Obesogenic Environment Scale; CFS, Chalder fatigue scale; Self-reported regular sleep schedule (0 = Irregular, 1 = Regular), Regular physical activity (0 = No, 1 = Yes), Sex (0 = Female, 1 = Male).

## Discussion

Among those with high levels of chronic fatigue, the proportion of women, individuals who do not exercise regularly, those who spend more than 4 h a day in sedentary activities, and those with irregular sleep schedules is higher. The literature has reported that chronic fatigue is predominantly associated with women and has attributed this to hormonal, cultural, and socio-economic differences (13, 23). Additionally, previous studies have reported that, in line with our findings, individuals with chronic fatigue have reduced participation in physical activity and an increase in sedentary behaviors and complaints of insomnia (24, 25). This situation is explained by changes in autonomic activity, alterations in hormone profiles, and the individual's behavioral and psychological responses (26, 27).

In our study, individuals with chronic fatigue appeared to be disadvantaged in terms of physical, social, and economic factors that support regular physical activity and healthy nutrition, while they seemed to be advantaged in terms of cultural factors and access to specialists. This can be interpreted as benefiting individuals with chronic fatigue from Turkish cultural traditions of nutrition and physical activity and from experts, but with a continued lack of social support and environmental disadvantages. Turkey has a rich culinary culture and a tradition of preparing meals at home, both of which support healthy eating (28). However, in Turkey, the culture of physical activity is not as prominent as the culinary culture. Although opportunities for physical activity have increased, an active lifestyle has not yet become a widespread social habit. Individuals experiencing chronic fatigue may be inclined to view culturally accepted healthy eating and physical activity as potential solutions, and they may seek expert advice to support these choices. In a previous study, it was stated that awareness of chronic fatigue may shape individuals' help-seeking behaviors and lifestyle attitudes (29). In another study, it was reported that individuals experiencing chronic fatigue are more likely to adopt a healthy lifestyle compared to healthy individuals (30).

We assessed the relationship of physical environment, social, and economic factors with fatigue holistically using a single scale in our study; in contrast, the literature generally analyzes these relationships separately. One study concluded that pocket parks alleviate mental fatigue among young adults, and that considering environmental features such as plant diversity, vegetation density, and the comfort of resting spaces in the planning and design process is important for enhancing these restorative effects (31). Other studies have found that both screen time and long working hours are associated with mental fatigue (32, 33). Another study, having a low household income was associated with a 53% higher risk of developing chronic fatigue (34). A systematic review has indicated that individuals with chronic fatigue can have multiple physical and psychological needs and often seek support from family, friends, and professionals to cope with the limitations in their daily lives (35). Nonetheless, it should be emphasized that the scale employed to assess obesogenic factors in our study does not encompass environmental pollutants that could influence chronic fatigue. It has been reported that environmental pollutants, known as xenobiotics (e.g., pesticides, industrial chemicals, heavy metals), may contribute to the development of chronic fatigue later in life by impairing immune system function, particularly during early life (36). Other studies have reported that environmental factors such as air, water, noise, and light pollution can also harm cognitive and psychological health (37, 38). Environmental pollution and pollutants are recognized as part of the obesogenic environment. Therefore, it is recommended to develop comprehensive scales in the future to jointly assess environmental pollutants and other obesogenic factors.

Nutrition directly affects the balance of the gut microbiota, the level of oxidative stress, and mitochondrial functions. Disruptions in these physiological processes can lead to chronic inflammation in the body and impairments in energy metabolism, contributing to the development of chronic fatigue syndrome (39). In this study, among individuals with high levels of fatigue, the proportion of those consuming fast food and frequently eating in front of screens increased, while the proportion of those who believed they were eating healthily decreased. Previous studies have also reported that the proportion of individuals with chronic fatigue syndrome who consume unhealthy foods is high and that improving diet quality can alleviate symptoms (30, 40).

In this study, the variables predicting chronic fatigue were examined. Accordingly, age, sex, BMI, and regular exercise did not play a significant predictive role. However, previous studies have shown significant relationships. Female sex, increased body mass index, and inactivity have been reported as risk factors for chronic fatigue (41, 42). Although inactivity is recognized as a risk factor in the literature, several studies have reported that the response to exercise differs in patients with chronic fatigue and that exercising can worsen symptoms (43, 44). While the age variable is not associated with chronic fatigue in line with our findings, it has been reported that increasing age is strongly associated with an increased risk of severe fatigue (45). Despite the significant differences in sex and regular exercise participation according to fatigue levels observed in the descriptive analyses, these variables did not emerge as predictors of chronic fatigue in the regression model. Even though key variables were incorporated into the regression model, the lack of a significant association might be attributed to complex interrelations among these variables or the exclusion of certain influential factors. In this study, variables such as educational level, socioeconomic status, and area of residence, which are likely to be associated with chronic fatigue and may act as confounding factors, were not included in the survey. It is recommended that future studies collect and incorporate these variables into the model. Our finding that sleep schedule is a determinant of chronic fatigue is supported by the literature (46–48). It has been reported that sleep problems may arise in individuals with chronic fatigue due to changes in cytokine profiles (46). In another study, it was determined that individuals with chronic fatigue are more sensitive to sleep delays and disruptions in sleep quality associated with low physical activity levels (47). While sleep disorders manifest as a symptom in individuals with chronic fatigue, they are also considered a contributing factor to the persistence of the condition. It has been stated that the deterioration of sleep quality can lead to a cycle that perpetuates chronic fatigue due to the interactions of biological, psychological, and social factors (48).

Significant correlations were found among all the scales used in the current study. Accordingly, the obesogenic environment, hedonic eating, mental fatigue, and physical fatigue are interconnected. Additionally, the obesogenic environment and hedonic eating are among the predictors of chronic fatigue. Although the findings do not directly establish causality, the existing literature provides biological mechanisms that could support these relationships. It has been reported that the obesogenic environment leads to low-grade chronic inflammation (3, 49). It has been suggested that chronic inflammation can increase the feeling of fatigue, along with cellular and structural changes in the central nervous system (50). Another study mentioned that prolonged hedonic eating behavior could alter the homeostatic system and lead to metabolic issues (10). Metabolic dysfunctions are seen as a possible mediator of chronic fatigue symptoms (51). Currently, food intake is more strongly influenced by the rewarding qualities of food than by actual hunger. Sensory cues related to food, including texture, smell, appearance, and taste, are intensively processed by the central nervous system, influencing the brain areas involved in food reward (52). The literature suggests that chronic fatigue syndrome and hedonic appetite share certain mechanisms, particularly those involving dysfunction in the brain's reward system. One study found reduced activity in the basal ganglia among individuals with chronic fatigue syndrome, which was associated with their fatigue (53). The basal ganglia are a group of structures deep in the forebrain that play a key role in reward mechanisms. Different nuclei within the basal ganglia, such as the nucleus accumbens, caudate, putamen, and globus pallidus, are involved in different types of reward. Food rewards have been reported to be processed more prominently in the basal ganglia of the right hemisphere (54). Another study found that the extent of changes in the right putamen, pallidus, and caudate was linked to levels of fatigue and motivation (55). The shared mechanisms between food reward and chronic fatigue pave the way for similar treatment approaches. Indeed, neuromodulation methods such as transcranial magnetic stimulation have been reported to be effective in both of these conditions (56, 57).

### Strengths and limitations

In this study, which focuses on a topic that has been little researched in the literature, the association between chronic fatigue and both hedonic appetite and obesogenic environments was examined. The evaluation of environmental and behavioral factors as predictors of chronic fatigue enhances the originality and scientific value of the study. However, it has a few limitations. First, the design of the research prevents the establishment of cause-and-effect relationships between the variables. For causal inferences, longitudinal or experimental designs should be preferred in future research. Secondly, a wide audience has been reached through the online survey method, but there are risks to the accuracy of the responses. There may be misunderstandings of the questions and random answering in the responses. Risks have been attempted to be mitigated with closed-ended questions containing clear and simple expressions and anonymity. Thirdly, the snowball sampling method may have led to homogeneity in the sample. Given that those who are more active on social media or more closely linked to the initial participants had a higher chance of being included in the sample, it is possible that the sample is relatively homogeneous in terms of demographic, socioeconomic, or behavioral features. Thus, the results may not be entirely generalizable to the whole adult population in Turkey. Future studies employing probability-based sampling methods are required to increase the generalizability of the results. Fourthy, the lack of data on participants' residential status and educational background can be considered a limitation of the study, as these factors may be related to obesogenic environmental risk. Another limitation of the study is that the data is based on the participants' self-reports. Due to recall bias, participants may have reported their heights and weights inaccurately or incompletely. Additionally, due to social desirability bias, they may have tended to reflect their lack of control over behaviors, environmental challenges, and levels of fatigue more positively. Future studies can improve the interpretation of findings by supporting self-report scales with objective measurements. Clinical interviews and laboratory biomarkers can be used to identify symptoms of chronic fatigue. In addition, Global Positioning System (GPS)-based digital tracking methods may help assess hedonic appetite, the obesogenic environment, and the interaction between them.

## Conclusion

According to the results of this study, among Turkish adults with a high perception of fatigue, there is a higher proportion of women, those who do not exercise regularly, those who spend more than 4 h a day in sedentary activities, and those with irregular sleep hours. Additionally, among individuals with a high level of fatigue, the proportion of those consuming fast food and frequently eating in front of a screen has increased, while the proportion of those who believe they eat healthily has decreased. Obesogenic environment, self-reported sleep schedule, and hedonic appetite are determinants of fatigue perception in adults. Additionally, the findings indicate that individuals with chronic fatigue face physical, social, and economic disadvantages in maintaining healthy eating and regular physical activity, and that environmental improvements are needed.

Our results reflect the need for interventions at both the individual and policy levels in the fight against chronic fatigue. At the individual level, to control the obesogenic environment, healthy foods should be kept at home, a sedentary lifestyle should be avoided, and the use of technology and time spent in front of screens should be limited. To manage hedonic appetite, a distinction should be made between emotional hunger and physiological hunger. Psychotherapy support can be sought to effectively combat emotional triggers that increase food intake. Another key factor of chronic fatigue, sleep schedule, can also be improved with various strategies that can be applied at the individual level. Sticking to regular sleep hours, limiting screen use before bed, and ensuring the room is quiet and dark can be effective in reducing levels of fatigue.

At the political level, exposure to an obesogenic environment can be reduced through urban planning. Active living can be supported by increasing green spaces, bike paths, walking, and recreation areas. Additionally, the proliferation of fast-food chains can be controlled in urban planning processes. During nighttime rest hours, noise control and appropriate lighting practices should be observed in cities. Policymakers should be aware of their critical roles in urban planning while addressing sleep schedule and the obesogenic environment in the fight against chronic fatigue. Additionally, in addition to improving physical conditions, initiatives should be undertaken to strengthen social interaction and support networks.

Chronic fatigue is a multifaceted public health issue. Preventing and managing it can positively impact social wellbeing in a multidimensional way by strengthening societal resilience in the long term, beyond just improving individuals' overall health. Since there may be many unidentified predictors of chronic fatigue, this area is open to further scientific research.

## Data Availability

The raw data supporting the conclusions of this article will be made available by the authors, without undue reservation.
